# Impact of Ilizarov Fixation Technique on the Limb Functionality and Self-esteem of Patients with Unilateral Tibial Fractures

**DOI:** 10.7759/cureus.5923

**Published:** 2019-10-16

**Authors:** Adeel A Siddiqui, Faiza Siddiqui, Masharib Bashar, Mariyam Adeel, Irfan Muhammad Rajput, Muhammad Soughat Katto

**Affiliations:** 1 Orthopaedic Surgery, Dow University of Health Sciences, Karachi, PAK; 2 Anatomy, Liaquat College of Medicine and Dentistry, Karachi, PAK; 3 Internal Medicine, Dr. Ruth KM Pfau Civil Hospital, Karachi, PAK; 4 Orthopaedics, Dow University of Health Sciences, Karachi, PAK; 5 Orthopaedic Surgery, Dow University of Health Sciences/ Dr. Ruth KM Pfau Civil Hospital, Karachi, PAK

**Keywords:** ilizarov fixation, limb functionality, self-esteem, tibial fractures, lower extremity functional scale, rosenberg self-esteem scale

## Abstract

Introduction

Tibial fractures with nonunion are frequently managed with Ilizarov external fixation. Living with an external frame has some psychological impact which is readily neglected from the literature. We conducted a study to evaluate the status of limb functionality in patients managed with the Ilizarov external ring fixator technique and assess their self-esteem while living with the frame.

Materials and methods

This is a prospective observational study conducted in the Orthopedic Department of Dr. Ruth KM Phau Civil Hospital, Karachi, Pakistan, from June 2018 to June 2019. A total of 26 patients consecutively managed with Ilizarov external fixation for infected nonunion, with unilateral tibial fractures, were included. To assess the postoperative functionality status, lower extremity functional scale (LEFS) was used. To assess and evaluate the impact of the external frame application on the self-esteem of these patients, Rosenberg's self-esteem (RSE) scale was used. For each patient, LEFS and RSE scales were administered at the time of hospital discharge, after six months of frame application, and at the time of removal of the frame.

Results

The mean duration of hospital stay was 4.11 ± 1.23 weeks. The mean LEFS scores increased by 47% from hospital discharge until the time of frame removal. The differences were highly significant (*p* < 0.001). There was a 12% decline in the mean score of self-esteem from the time of discharge till the time of removal of the Ilizarov frame and these differences were highly significant (*p* < 0.001).

Conclusion

Ilizarov technique improves the limb functionality status significantly in participants with a unilateral tibial fracture. However, it also reduces their self-esteem during the period of frame application. Psychological support is recommended for participants living with an external fixation frame to protect their self-esteem.

## Introduction

In general, the third most common long bone injury is a fracture of the tibia. Most tibial fractures are distal and few of them are open [1]. However, the risk of malunion (17%), nonunion (7.5%), infection (10%), and loss of reduction (32%) is high [2-5]. The treatment options for tibial fractures include casting, nailing, plating, and external fixation with either a circular frame or a mono-lateral frame. High-energy traumatic injuries with associated soft tissue and skeletal injuries present management challenges. Tibial fractures and their non-union are frequently reported in high-energy traumatic injuries to the lower limbs [1,6-7].

Tibial nonunion is associated with a higher risk of persistent contamination, subsequent bone loss, deformity, a discrepancy in leg length, and stiffness of joints. In such cases, a multi-faceted approach has to be adapted. It must include one reunion, control of infection, and reverting functional independence of the limb (through debridement, flaps or grafts, use of antibiotic beads, bone grafting, and even bone transport) if needed [8]. These procedures have to be stepped, must be closely monitored, and are associated with prolonged recovery time and delayed rehabilitation [9].

Ilizarov technique is based on distraction osteogenesis. It tackles all of the above-discussed issues with tibial nonunion simultaneously. It enables regeneration of large bony defects, removes infected nonunion, provides stable fixation, and allows early weight-bearing and mobility [6,8].

Although the functional outcome with the Ilizarov ring fixator has been established in patients with tibial fractures, there has been little focus on the psychological aspects of living with the frame. We conducted this study to evaluate the status of limb functionality in patients managed with the Ilizarov technique and assess their self-esteem while living with the frame.

## Materials and methods

This is a prospective observational study conducted in the Orthopaedic Department of Dr Ruth KM Phau Civil Hospital, Karachi, Pakistan, from June 2018 to June 2019 (until the last follow-up) in which 26 patients of age 17 to 65 years were admitted and consecutively managed with Ilizarov external fixator for infected nonunion tibial fractures. These patients were followed throughout their hospital stay and afterwards until the removal of their external Ilizarov frame. The study was approved by the institutional review board and consent of the guardian/assent of the patient was taken. Patients with polytrauma, concomitant neurovascular injury, and intra-articular injury were excluded.

A structured questionnaire was constructed. It included the patient’s age, gender, cause of injury, duration of fracture (old versus acute), and location of fracture (proximal, middle, distal tibia). The fractures were graded according to the AO-Müller/Orthopaedic (AO) classification [10]. Open fractures were classified according to the Gustilo Classification [11]. Union time, time to partial and full weight-bearing, and duration of application of the Ilizarov frame were also recorded for all patients.

To assess the postoperative functionality status, lower extremity functional scale (LEFS) was used in this study. It is a 20-item score that measures physical function. For each item, there is a five-point Likert score (zero-four) with lower scores depicting a greater degree of functional difficulty. The minimum score is zero and the maximum is 80. The function is defined as 0-19, extreme difficulty or unable to perform the activity; 20-39, quite a bit of difficulty; 40-59, moderate difficulty; 60-79, little difficulty; and 80, no difficulty. The reliability score of LEFS has been stated in the literature (0.94) [12-13].

To evaluate the impact of the external frame application on the self-esteem of these patients, Rosenberg’s self-esteem (RSE) scale was used. It is a 10-item score that assesses positive as well as negative feelings about the self. All items are responded on a four-point Likert scale (1-4) ranging from strongly agree to strongly disagree [14]. For each patient, the LEFS and RSE scales were administered at the time of hospital discharge, after six months of frame application, and at the time of removal of frame application.

Data were entered and analyzed using SPSS v. 22 (IBM Corp., Armonk, NY, US). The mean and standard deviation were calculated for all continuous variables, while frequency and percentages were calculated for categorical variables. A dependent *T*-test was applied to establish a statistical correlation between the mean LEFS and RSE score at the time of discharge and at the time of external frame removal. The mean of the percentage change from discharge to frame removal was also calculated. *P*-value ≤0.05 was taken as significant.

## Results

Twenty-six consecutive patients with unilateral tibia fractured fulfilling the inclusion criteria were surgically managed using the Ilizarov method of external fixation osteosynthesis. There were 19 (73.1%) male and seven (26.9%) female participants. Their mean age was 43 ± 4 years. The injury was caused as a result of road traffic accidents in 13 (50.0%) patients, fall in 11 (42.3%) patients, and two (7.7%) patients fractured their tibia as a result of a gunshot injury. There were 15 (57.7%) closed fractures and eight (30.8%) open fractures. Out of these eight open fractures, two (25.0%) were grade I, four (50.0%) were grade II, and two (25.0%) were grade IIIB fractures. In 11 (42.3%) patients, the right tibia was involved and in 15 (57.7%) patients, the left tibia was involved. According to AO classification, there were four (15.4%) 41A type fractures, two (7.7%) 41B and 41C type each, five (19.2%) 42A type fractures, seven (26.9%) of 42B type, three (11.5%) were 42C, one (3.8%) was 43A, and two (7.7%) were 43C type fractures. There were 14 (53.8%) acute fractures and 12 (46.2%) old fractures. There were six (23.1%) fractures in the proximal tibia, seven (26.9%) in middle, and 13 (50.0%) in the distal tibia.

The mean union time was 18.2 ± 3.4 weeks (range: 10-55 weeks). The mean time to partial weight-bearing walking was 3.87 ± 1.12 days (range: 1-20 days). The mean time to full weight-bearing walking with range was 28.43 ± 2.55 days (range: 5-134 days). All means were calculated from the day of application of the Ilizarov external fixator.

The mean duration of hospital stay was 4.11 ± 1.23 weeks (range: 1.12-19.30 weeks). The mean duration of the application of Ilizarov external fixator was 11.35 ± 2.45 months (range: 10-18 months). For each patient, the LEFS and RSE scales were administered at three instances: at the time of discharge, after six months of frame application, and at the time of frame removal. Table 1 shows LEFS scores at three intervals. The mean LEFS scores increased by 47% from hospital discharge until the time of frame removal. The differences were highly significant.

**Table 1:** Mean lower extremity functional scale score at the time of discharge, after six months, and after removing Ilizarov frame SD; standard deviation *Comparison was done between at the time of hospital discharge and after removing the Ilizarov frame.

Time Intervals	Lower extremity functional scale score		P-value*
	Mean ± SD	Mean change (%) from discharge to frame removal	
At the time of hospital discharge	33 ± 7	47.6 ± 2.2	<0.0001
Six months follow-up	54 ± 11		
After removing the Ilizarov frame	63 ± 9		

Table 2 shows the mean RSE scale scores at three-time intervals. There was a 12% decline in the mean score of self-esteem from the time of discharge until the time of removal of the Ilizarov frame. The differences were highly significant.

**Table 2:** Mean Rosenberg self-esteem scale scores at the time of discharge, after six months, and after removing Ilizarov frame SD, standard deviation *Comparison was done between at the time of hospital discharge and after removing the Ilizarov frame.

Time Intervals	Rosenberg self-esteem scale score		P-value*
	Mean ± SD	Mean change (%) from discharge to frame removal	
At the time of hospital discharge	23.5 ± 1.13	12.7 ± 1.15	<0.0001
Six months follow-up	22 ± 2		
After removing ilizarov frame	20.5 ± 1.0		

## Discussion

This study has shown striking results. By the time of external frame removal, this study showed a 50% reversal in limb functionality. However, there has also been a 12% decrease in the overall self-esteem of these participants over time. Nonunion of the tibia that is infected causes various problems such as deformity, bone loss, infections, and severe impact on health-related quality of life [15]. The impact of tibial shaft fracture nonunion on the physical and mental health of the patient was severe and can be compared to arthrosis at end-stage and even more severe than that of congestive heart failure [15]. This poses a serious challenge for the orthopaedic surgeons, and the Ilizarov method has been successful in solving all the associated problems. Although various studies have reported a high success rate of Ilizarov in non-union fractures, pain, long treatment process, and prolonged external fixation can be considered as the major limitations of the technique [16].

Quite remarkably, this study has focused on the psychological impacts of external fixation, which are neglected in Pakistani literature. However, this study has its limitations too. It is a single-centre study with very small sample size; hence, the results cannot be generalized. Furthermore, the patients were only followed until the removal of the external frame, and hence, the authors cannot state whether or not self-esteem reverted in the months after frame removal.

In our study, while the lower extremity function limb score increased, self-esteem score reduced. Wang H et al. in 2017 in his study including 15 patients stated that mental capacity score and physical capacity score increases with time [17].

We recommend large-scale randomized trials to assess the impact of external fixation on self-esteem and the level of functionality in these patients. We also recommend concomitant psychological support to protect the self-esteem of these participants living with an external frame. It is also recommended to conduct such longitudinal studies that can follow up the patients for a good period even after the removal of the frame to study the time taken for the self-esteem score and the level of functionality of these participants to revert to normal. 

## Conclusions

This study has concluded that the Ilizarov technique improves the limb functionality status significantly in participants with unilateral tibial fractures. However, it also reduces their self-esteem during the period of frame application. Psychological support is recommended for participants living with an external fixation frame to protect their self-esteem, which, if not reverted after frame removal, can have debilitating impacts on their mental well-being.

## References

[1] Thompson JH, Jahangir A (2019). Tibia Fractures Overview. StatPearls.

[2] Goodbody CM, Lee RJ, Flynn JM, Sankar WN (2016). Titanium elastic nailing for pediatric tibia fractures: do older, heavier kids do worse?. J Pediatr Orthop.

[3] Hope P, Cole W (1992). Open fractures of the tibia in children. J Bone Joint Surg Br.

[4] Irwin A, Gibson P, Ashcroft P (1995). Open fractures of the tibia in children. Injury.

[5] Shore BJ, DiMauro JP, Spence DD, Miller PE, Glotzbecker MP, Spencer S, Hedequist D (2016). Uniplanar versus taylor spatial frame external fixation for pediatric diaphyseal tibia fractures: a comparison of cost and complications. J Pediatr Orthop.

[6] Bakhsh K, Atiq-Ur-Rehman FK, Mohammad E, Ahmed W, Saaiq M (2019). Presentation and management outcome of tibial infected non-union with Ilizarov technique. Pak J Med Sci.

[7] Khan MS, Rashid H, Umer M, Qadir I, Hafeez K, Iqbal A (2015). Salvage of infected non-union of the tibia with an Ilizarov ring fixator. J Orthop Surg.

[8] Yaqoob U, Nadeem Z, Mehmood M (2016). Descriptive pattern of fractures among motor bike riders presenting in jinnah postgraduate medical centre, karachi. Br J Med Med Res.

[9] Sen C, Eralp L, Gunes T, Erdem M, Ozden VE, Kocaoglu M (2006). An alternative method for the treatment of nonunion of the tibia with bone loss. J Bone Joint Surg Br.

[10] Müller ME, Nazarian S, Koch P, Schatzker J (1990). The Comprehensive Classification of Fractures of Long Bones.

[11] Gustilo RB, Anderson JT (1976). Prevention of infection in the treatment of one thousand and twenty-five open fractures of long bones: retrospective and prospective analyses. J Bone Joint Surg Am.

[12] Binkley JM, Stratford PW, Lott SA, Riddle DL (79). The lower extremity functional scale (LEFS): scale development, measurement properties, and clinical application. 1999.

[13] Schep NW, van Lieshout EM, Patka P, Vogels LM (2009). Long-term functional and quality of live assessment following post-traumatic distraction osteogenesis of the lower limb. Strategies Trauma Limb Reconstr.

[14] Rosenberg M (1965). Society and the Adolescent Self-image.

[15] Brinker MR, Hanus BD, Sen M, O'Connor DP (2013). The devastating effects of tibial nonunion on health-related quality of life. J Bone Joint Surg Am.

[16] Lavini F, Dall’Oca C, Bartolozzi P (2010). Bone transport and compression-distraction in the treatment of bone loss of the lower limbs. Injury.

[17] Wang H, Wei X, Liu P (2017). Quality of life and complications at the different stages of bone transport for treatment infected nonunion of the tibia. Medicine.

